# Predictive and Mechanism-Based Toxicity Evaluation of Engineered Nanoparticles

**DOI:** 10.3390/nano16030185

**Published:** 2026-01-30

**Authors:** Rongrong Liu, Bing Yan

**Affiliations:** Key Laboratory for Water Quality and Conservation of the Pearl River Delta, Institute of Environmental Research at the Greater Bay Area, Ministry of Education, Guangzhou University, Guangzhou 510006, China; drbingyan@yahoo.com

The rapid expansion of nanotechnology has driven the widespread use of engineered nanomaterials in energy, electronics, medicine, and consumer products [1,2]. Inevitably, however, these materials are released into the environment during their production, use, and disposal, raising increasing concern about their potential ecological and human health risks [3,4]. Owing to their nanoscale dimensions, large specific surface area, and highly reactive surface chemistries, nanoparticles (NPs) interact with biological systems in ways fundamentally different from their bulk counterparts, often giving rise to novel bioavailability, transport, and toxicity profiles [5]. Consequently, the development of scientifically rigorous, reliable, and predictive frameworks for NP toxicity evaluation has become a prerequisite for the sustainable and responsible advancement of nanotechnology.

Nanotoxicology has evolved from early assessments of acute cytotoxicity toward a more comprehensive understanding of long-term, low-dose, and system-level biological effects. The contributions to this Special Issue reflect this transition and illustrate important progress on multiple fronts. With respect to toxicological outcomes, it is now evident that NP toxicity is strongly dependent on both material properties and exposure context. For example, carbonaceous nanoparticles—a major component of fine atmospheric particulate matter—are closely associated with adverse effects on the respiratory, cardiovascular, and nervous systems [6]. Likewise, silver nanomaterials exhibit pronounced shape-dependent toxicity, with spherical, cubic, and prismatic forms eliciting markedly different biological responses [7].

In parallel, this Special Issue highlights the diversification and increasing sophistication of toxicity assessment models. Studies span conventional in vitro systems (e.g., A549 and HepG2 cells) to whole-organism models, including rats, zebrafish, rainbow trout, and aquatic plants such as *Lemna minor* L. [7,8,9,10,11]. Importantly, model choice has emerged as a critical determinant of toxicological outcomes. Differences in nanoparticle uptake, intracellular processing, and stress responses between primary cells and immortalized cancer cell lines underscore the necessity of selecting biologically relevant systems [12]. Moreover, the integration of experimental data with computational nanotoxicology approaches offers a powerful strategy for screening and prioritizing emerging nanomaterials [13].

Despite these advances, several fundamental challenges remain. First, the intrinsic heterogeneity of NPs, encompassing their size, shape, surface chemistry, and colloidal stability, combined with highly variable exposure conditions severely limits cross-study comparability and predictive extrapolation. For example, the toxicity of MXenes is closely linked to their environmental stability and degradation pathways [14]. Second, the accurate identification, quantification, and speciation of NPs and their transformation products within complex biological matrices remain technically demanding, as illustrated by the in situ reduction of Ce(IV) to Ce(III) in plant tissues following CeO_2_ NP exposure [10]. Third, although oxidative stress, inflammatory signaling, and endocrine disruption are frequently implicated, a mechanistically integrated and system-level understanding of the effects of NP-induced toxicity—such as TiO_2_ NP-mediated impairment of zebrafish gonadal function—has yet to be established [11].

In future, nanoparticle toxicity evaluation must transition toward more precise, mechanism-based, and predictive paradigms. This will require the systematic integration of experimental toxicology with advanced computational tools, including QSAR, machine learning, and deep learning models, to enable intelligent screening, hazard ranking, and exposure-effect prediction. Equally important, risk assessment frameworks must adopt a life-cycle perspective, accounting not only for pristine nanomaterials but also for their environmental transformation products and dynamic behavior in complex biological and ecological systems [15]. Only through such integrative and forward-looking approaches can society fully realize the benefits of nanotechnology while ensuring the protection of human health and environmental safety.
